# Stakeholder perspectives on transferability of a 12-week physical activity and sedentary behaviour intervention for ethnically diverse community dwelling older adults: a qualitative study

**DOI:** 10.1136/bmjopen-2025-107380

**Published:** 2026-04-24

**Authors:** Naureen Akber Ali Meghani, Joanne Hudson, Gareth Stratton, Jane Mullin

**Affiliations:** 1 Department of Applied Health Sciences,College of Medicine and Health, University of Birmingham, Birmingham, UK; 2 Applied Sports Technology, Exercise and Medicine (A-STEM) Research Centre, Swansea University, Swansea, Wales, UK

**Keywords:** Aging, QUALITATIVE RESEARCH, Primary Prevention, Primary Care

## Abstract

**Introduction:**

Numbers of ethnically diverse older adults are increasing in the UK. These individuals often have complex health problems that are exacerbated by language barriers (ie, limited English proficiency), acculturation experiences and socio-economic level. Further, this diverse group is also the most sedentary and least active subgroup in the wider population which raises major health issues. A number of interventions have been implemented to improve older adults’ physical activity and decrease their sedentary behaviour. Nevertheless, there is a lack of research examining how stakeholders’ perspectives can inform the transferability of interventions into the real-world particularly for ethnically diverse older adults. Therefore, the purpose of the current study was to explore the perspectives of stakeholders regarding the transferability of a 12-week intervention that aims to increase activity and decrease sedentary behaviour among ethnically diverse sedentary older adults.

**Methods:**

A qualitative exploratory study employing reflexive thematic analysis was conducted using purposive sampling and in-depth interviews to recruit a diverse group of stakeholders representing varied professional roles, service delivery and organisational sectors related to older adults’ physical activity and well-being. The Population–Intervention–Environment–Transfer Model of Transferability (PIET-T) model served as a theoretical and conceptual framework for assessing the transferability of health interventions. Prior to the interview, the researcher explained the intervention study that was assessed in a prior feasibility study. This helped us identify stakeholder perspectives about potential challenges and practical considerations for transferring the intervention within existing policy and service frameworks. The primary researcher (NAAM) transcribed data from recorded interviews. Using reflexive thematic analysis, themes were generated from the data set and were interpreted using the PIET-T model.

**Findings:**

The findings showed that different concepts of the PIET-T model influenced intervention transferability. The findings generated the following key themes: (1) User-centred and organisation supported programmes (Population), (2) intervention appropriateness and adaptations (Intervention), (3) organisational and system context (Environment) and (4) transferability and implementation factors (Transferability). Overall, the PIET-T model addresses key factors within each domain to facilitate the transferability of intervention.

**Conclusion:**

Exploring diverse stakeholder perspectives was crucial for facilitating transferability and ensuring readiness for real-world implementation. Stakeholders suggested key modifications, including translated materials, adjusted duration, flexible digital delivery options and stronger collaboration with local organisations and healthcare systems to improve transferability among ethnically diverse older adults.

**STRENGTHS AND LIMITATIONS OF THIS STUDY**

- A well-established reflexive thematic analysis approach was used to understand in-depth stakeholders’ perspectives about the transferability of the intervention.
- Given the exploratory focus, we included diverse stakeholders to capture varied perspectives; however, findings from under-represented groups should be interpreted with caution.
- The Population–Intervention–Environment–Transfer Model of Transferability model allowed for deeper exploration and provided rich insights in assessing and guiding the intervention transferability.
- The interviewer is also the intervention designer, which might result in social desirability bias; however, the researcher tried to minimise bias by assuring participants that no personally identifiable information would be displayed or published.
- Due to time and resource constraints, older adults were not included in this transferability study. However, they will be interviewed in a larger effectiveness trial to assess the intervention’s real-world transferability.

## Introduction

 The UK government makes significant investments to address older adults’ low activity level and high sedentary trends through national initiatives and funding programmes.^1^,^5^ However, the programmes currently implemented do not present convincing evidence that they are effective in raising activity levels and reducing sedentary behaviour among ethnically diverse older adults.^1^ This may be because prior research has not focused on home-based activities, but instead on activities that take place outside the home, which may pose a number of obstacles for ethnically diverse older adults, such as financial challenges, social responsibilities, language barriers (ie, limited English proficiency), low confidence level, limited resources and restrictions based on culture and religion.^1^ Further, lack of key stakeholders’ involvement in programme design can undermine intervention effectiveness, whereas involving various stakeholders offers a wider understanding of barriers and facilitators from diverse perspectives, informing more meaningful long-term interventions.^5 6^

Furthermore, it has been noticed that sedentary and inactive older adults consider high-intensity activity to be unsuitable and that only meaningful activities should lead to physical activity, like household chores.^7^ As even a small increase in activity among sedentary older adults might have positive health impacts,^8^,^10^ this emphasises the necessity of incorporating innovative and practical interventions for older adults that incorporate these considerations.^11^ Given this, our 12-week home space intervention employed a brief health coaching session, a pamphlet, weekly reminder messages and a wearable activity tracker to optimise older adults’ home space in order to increase their physical activity and reduce sedentary behaviour. Despite a preliminary feasibility trial confirming that the intervention is appropriate for use with older adults, it is currently unknown whether the home-based physical activity and sedentary behaviour intervention is transferable to real-world contexts and can be used as part of current initiatives to enhance older adults’ health and well-being. Transferability of an intervention is taken as the extent to which an intervention’s impact in one context can be seen in another,^12^ which is crucial for improving health. Health promotion interventions incorporate action that targets behaviour modification, social and physical environment changes and public policy alterations. They provide individuals with important resources to maintain and improve their own health.^13^,^15^ These interventions are complex^16^,^18^ and the link between the intervention and the setting in which they occur is also complex.^13 19^ One of the most crucial factors to take into account before implementing an intervention into practice is the intervention outcome consistency when transferred from one context to another, or from research to the real-world.^13^ In health promotion, achieving this might be difficult. Although an intervention that has proven successful in one setting may still be beneficial in other settings, its effects may differ from those of the original intervention.^20^ Hence, stakeholder involvement is essential to increase the likelihood that the intervention will be implemented successfully in real-world contexts.^21^

Stakeholders are individuals or organisations that are responsible for or affected by health and healthcare decisions,^22^ and many funding agencies view their participation in research as crucial to guide these interventions.^23^ Involvement of stakeholders and stakeholder organisations can provide important information about the possible role and capacity of organisations in implementing the intervention (such as funding, referring), identify barriers and facilitators to integrate the intervention into routine service delivery and suggest strategies (including recommended intervention modifications) to ensure the intervention is sustainable and transferable to a real-world setting.^21^ However, this important stage is rarely conducted by researchers, and as a result, there is presently a lack of information that provides stakeholder perspectives to inform the intervention’s transferability.^21^ Glasgow and colleagues^24^ state that most interventions are developed and assessed effectively, but many of them ‘get lost in translation’ because they are not applied sustainably in the different ‘real life’ conditions for which they are intended. To promote long-lasting gains in older adults’ physical activity and sedentary behaviour, stakeholders should be heavily involved in the implementation and maintenance of interventions following the conclusion of research programmes that have delivered and assessed these interventions.^25 26^

Examining transferability is crucial to implement the intervention into action.^13^ In this sense, stakeholder perspectives are a useful and productive source of information^26 27^ since they provide a distinct perspective on the intervention, which is crucial to comprehending its transferability. Their opinions on the intervention actually influence whether they decide to replicate or transfer it, and if so, how they alter it to ensure intervention adaptability and sustainability in a real-world situation.^13^ In that regard, the 12-week home space intervention that the researcher designed^28^ and implemented with ethnically diverse older adults was presented to stakeholders to explore their perspectives on its transferability.^29^

## Methods

### Study design

The study employed an explorative qualitative research design and was conducted using purposive sampling and semistructured in-depth interviews with stakeholders from 30 July 2024 to 30 September 2024 to explore stakeholders’ perspectives regarding the transferability of the intervention into their contexts. Semistructured in-depth interviews are particularly effective to examine participants’ perspectives and feelings in circumstances when a comprehensive understanding of the condition and context is essential.^30^ Given the complex nature of assessing the intervention transferability, this qualitative approach offers a strong foundation for gathering detailed information from stakeholders.^30^ Guidelines for qualitative research included in the consolidated checklist (online supplemental file 1, Checklist), were used for reporting this study.

### The 12-week intervention

The interview is based on the multicomponent home-based intervention to increase activity and reduce sedentary behaviour in older adults, which has been tested for feasibility and acceptability. Supported by the social ecological model^31^ and the habit-based model, the intervention encompasses: (1) encouraging older adults to optimise their home space for activity (environmental level); (2) offering individualised face-to-face health coaching sessions and pamphlets to raise awareness of sedentary behaviour and physical activity and their effects on health (individual level); and (3) using wearable activity trackers and reminder messages to give them a cue or prompt to switch from prolonged sitting to standing, stepping or light activity in their home setting to modify their behaviour (individual level). The details of the intervention are mentioned in prior published paper^28^ and TIDieR (Template for Intervention Description and Replication) checklist (online supplemental file 2, Checklist) for information regarding intervention is provided

### Population–Intervention–Environment–Transfer Model of Transferability

The Population–Intervention–Environment–Transfer (PIET) model serves as a theoretical and conceptual framework for assessing the transferability of health interventions and can assist in making decisions on intervention transferability (table 1). The population, the intervention, the environment in the primary and target settings, and the intervention’s transference are the four essential components required for this transferability.^32^ It focusses on the viewpoint of the decision-makers who represent stakeholders, aiming to improve the target population’s health status (P) and considering the transfer of an intervention (I) from a primary context to a target context. In this study, the primary context refers to the initial feasibility study in which the intervention was developed and implemented, while the target context refers to the settings in which stakeholders may apply the intervention. Decision-makers may involve different academics, professionals, legislators, stakeholders and organisational leaders.

**Table 1:** Operationalisation of PIET-T domains in the study

PIET-T domain	Operational definition for this study	Methods/data sources
Population (P)	Ethnically diverse community-dwelling adults aged ≥65 years (male and female) who were sedentary (self-reported ≥5 leisure hours sitting/day), not meeting moderate-to-vigorous physical activity guidelines (>150 min/week) without physical or cognitive impairments that might limit participation.	Demographic survey, baseline health and activity assessment, interviews from participants.
Intervention (I)	12-week home-based physical activity programme including: healthcare coaching sessions, informational pamphlets, wearable activity (to remind participants to take breaks from prolonged sitting time) and reminder messages to increase activity and reduce sedentary time.	Intervention manuals, coaching session records, activity and sedentary level data.
Environment (E)	Home settings, cultural context, access to technology, local support from family.	Participant interviews.
Transfer (T)	Adapting and implementing the programme in the target population’s home environment; piloting of materials and delivery strategies; refinement based on participant and stakeholder feedback.	Implementation plan, stakeholder consultations and interviews to modify intervention.

The decision-making process may consider the opinions and requirements of the target population (P), as well as the coordinating team in the target environment (E), to plan, arrange and execute the transfer (T). It is assumed that the population, the intervention and the environment all have an impact on one another. The outcome is determined by the combination of these three constructs. To identify a suitable intervention, the health issue is examined using the (baseline) population characteristics of the target context. The decision-maker gathers data on the evidence presented in a primary context, such as the site, demographic features and research design. Before determining whether to transfer the intervention, the decision-maker must consider the conditions of both the primary context and the target context (his or her own setting). As a result, the intervention may need to be adjusted to fit the desired situation.

The transfer can occur at various levels in a target setting, such as the individual, organisational, local (community or region), national or even global levels. Outcome transferability is influenced by both the primary and target environments. Outcome transferability refers to the extent to which the intervention can achieve similar effects in the target context as observed in the primary context. The design of the transfer is guided by information from the primary context. Concurrently, as transferability also depends on how these three constructs interact, the target contexts must be taken into account when planning and executing the transfer. Therefore, the population, the intervention, the environment in the primary and target contexts, and the transfer procedure itself all have an impact on the transferability of the health interventions. This implies that for the outcomes of the target context to be similar to those of the primary context, the constructs in both contexts must be the same. This does not always imply that criteria, standards and procedures in the primary and target contexts must precisely coincide to ensure transferability. Transferability depends on the circumstances of each context, comparing the primary and target contexts is useful. Information from the target context as well as the primary context is needed for this. The differences and similarities between the two contexts must be identified in order to (1) assess whether and how (under what conditions) the intervention is suitable for improving the target population’s health and (2) organise the process of transfer systematically.^32^

### Study recruitment and setting

To facilitate the recruitment of stakeholders, regional coordinators and sub-coordinators of organisations with a formal remit for older adults’ health and well-being (eg, physical activity promotion or sedentary behaviour reduction) in South Wales were approached. Stakeholders who are engaged in the planning, developing and execution of programmes that support and improve the physical activity or health and well-being of older adults were identified and contacted. Through their contacts we reached out to numerous stakeholders who either directly or indirectly work with older adults using recruitment letters and posters/leaflets. Additionally, we used the intranet to advertise throughout the university and collaborated with their contacts to recruit through their physical activity, play, health and community networks.

Participants who were interested were contacted by the researcher by ‘phone or email’ and were sent a participant information sheet. The lead researcher also provided an explanation of the process of the study and answered any questions participants had. Participants were informed that taking part in the research was voluntary, and they had the right to withdraw at any point without incurring any penalties. They were also told to contact the research team if they no longer wanted to take part in the study. If the stakeholder was happy to be involved, they were provided with a consent form to sign before the interview. Participants were still able to contact the lead researcher with any questions or concerns.

### Study participants

The researcher recruited a diverse group of stakeholders (n=10) representing variation in professional roles, organisational sectors and service delivery contexts related to older adults’ physical activity and well-being. Stakeholders included programme officers/leads, healthcare professionals and government and non-government officers working across community services, professional associations and government departments. Given time and resource limitations, we were not able to incorporate older adults in the intervention’s transferability process, although we have previously conducted qualitative interviews to determine the intervention’s acceptability in a previous study.^33^

### Inclusion criteria

We targeted individuals whose work primarily focuses on older adults, even if they also contribute to work with other population groups. Therefore, we recruited:

- Individuals who are involved in setting agendas, monitoring funds and offering support and advocacy for health and well-being of older adults, including engagement in physical activity.
- Individuals who determine funding provision for the development, implementation and expansion of interventions to enhance the health and well-being of older adults through physical activity initiatives or fall risk reduction programmes.
- Individuals who may not have direct contact with participants but are indirectly involved in the planning and execution of various programmes aimed at improving older adults’ activity, well-being and sedentary behaviour intervention.
- Individuals who work directly with older adults and implement interventions.
- Individuals conducting research, such as pilot testing, feasibility studies, effectiveness trials and the evaluation of interventions suggested to enhance older adults’ health through physical activity promotion or sedentary behaviour reduction.

### Exclusion criteria

Individuals whose work primarily focuses on other population groups and not on older adults were excluded.

### Patient and public involvement

The public and patients were not involved in determining the research agenda.

### Data collection

Prior to the interview, the lead researcher explained the intervention study that was assessed in a prior feasibility study.^28^ This assisted us in understanding the potential difficulties or practical concerns of implementing this intervention on a larger scale within the framework of current policy and service. A semistructured interview guide was created to conduct in-depth interviews (online supplemental file 3; Interview tool guide) using the PIET Model of Transferability (PIET-T) model.^34^ This model can support decision making in this context, serving as a theoretical conceptual model for assessment of the health intervention’s transferability. The population, the intervention, the environment in the target and primary contexts, and the transference of the intervention are the four essential elements required for this transferability.^34^

The stakeholder interview questions were explicitly mapped to the PIET-T framework to guide assessment of transferability. In terms of population (P), stakeholders were asked whether the 12-week home-based intervention was appropriate for ethnically diverse, sedentary older adults aged ≥65 years, including its socio-cultural relevance and suitability for other older adult groups (eg, those in service delivery settings or care homes). In terms of intervention (I), questions explored the feasibility and appropriateness of the intervention components (health coaching, educational materials, wearable activity trackers and reminder messages), and how the simplicity or complexity of these components influenced their perceived suitability and potential use beyond the study context, with regard to the transferability of the findings. In terms of environment (E), stakeholders reflected on organisational and contextual factors affecting delivery. Finally, in terms of transfer (T), stakeholders were asked about the possibility of integrating the intervention into existing programmes, the support required for scale-up and anticipated facilitators and challenges to transferring and sustaining the intervention at regional/state and national levels. Each participant was given a unique ID number, which was used throughout the study. After explaining the intervention, the lead researcher carried out interviews with the stakeholders to determine their perspectives regarding the intervention’s transferability in their particular setting.

The interview was conducted by the primary researcher (NAAM), who is qualified and skilled in qualitative research and has a considerable amount of experience in carrying out interviews. To meet the needs and preferences of the stakeholders, both in-person and online interview options (via Teams or Zoom) were provided. Interviews were audio-recorded and lasted between 15–30 min in length with three in-person interviews and the remaining performed online using Zoom. This approach guaranteed flexibility and inclusivity, enabling participants to choose the mode that best suited their unique circumstances. Therefore, the hybrid technique used here is consistent with contemporary qualitative research methodologies that place an emphasis on flexibility and participant-centred data collection.^35^

We closely monitored the data collection process in compliance with best practices for qualitative research, starting data analysis at the same time as data collection and assessing whether any new themes or insights continued to emerge as we proceeded with the interviews. Interviews were conducted until sufficient depth and richness of data were obtained to support meaningful thematic development in line with Braun and Clarke’s.^36^

### Data analysis

The primary researcher (NAAM) transcribed data from recorded interviews. The interviewer’s field notes (NAAM) were assessed in conjunction with the interview transcripts to facilitate verification and cross-checking of the findings. Reflexive thematic analysis was conducted in accordance with the procedures outlined by Braun and Clarke.^37^,^39^ Reading interview notes and transcripts multiple times was the first step in becoming familiar with the data (Step 1). The initial coding process was used to create codes in accordance with the data (inductive coding) while the deductive coding process was informed by the PIET-T model’s concepts: Population–Intervention–Environment–Transfer Model of Transferability^32^ -(Step 2). Step 3 involved searching for themes and subthemes (eg, *intervention convenience* and *simple*) in order to create meaningful themes (like *practical intervention*). Following this, themes (like *feasible intervention*) were examined (Step 4) and grouped according to the PIET-T model’s elements (like *intervention appropriateness and adaptations—PIET-T model*; Step 5). The final step was to write up the findings (Step 6). Using an assigned number and their function (eg, stakeholder 1, government officer), anonymised written participant quotes were used to provide a concise summary of the main themes. JH, the second author, likewise coded two transcripts at the beginning of Step 2. There were no notable discrepancies in interpretation, although some slight differences in usage of terms. Thus, both NAAM and JH reviewed and assigned the final themes.

### Researcher reflexivity

The stakeholder interviews were conducted by NAAM, who has a background as an adult health nurse and experience in research on mental health, sedentary behaviour and physical activity in older adults. At the time of the study, she was a PhD researcher and was involved in the development of the 12-week home-based intervention under the supervision of JH and GS. This dual role as both interviewer and intervention developer may have introduced the potential for social desirability bias. To minimise this, participants were assured that responses would remain confidential and were encouraged to provide honest views, emphasising that there were no right or wrong answers. Interviews were conducted with participants who were not previously known to the researcher. JH, an experienced qualitative researcher and Health and Care Professions Council (HCPC)-registered psychologist, oversaw the research process to ensure rigour. Although coauthors were not involved in data collection or analysis, they contributed to interpretation and critical reflection during the analysis phase (Step 6).

### Dissemination

Confidentiality and anonymity were maintained throughout the study. Participants were informed about voluntary participation. The findings will be disseminated to research participants through seminars and workshops, as well as the current peer-reviewed journal publication.

## Results

15 stakeholders from different organisations that engage with ethnically diverse older adults were contacted to take part in the study. 10 stakeholders provided consent and participated. The remaining five did not participate: three declined due to organisational restructuring, one due to a health issue and one was unable to find an appropriate time for the interview.

The 10 interviewees were representative of nine stakeholder organisations that provided physical activity, health and well-being services. The stakeholder sample organisations involved community services/non-government organisations (n=7), professional associations (n=2) and government departments (n=1). The roles performed by participants were research officer, civil officer, government officer, programme lead and engagement officer. The average age of the participants was 45.2±8.2 years, with 60% of them being female (F) and 40% being male (M). To identify important stakeholder perspectives regarding the design and transferability of the 12-week intervention research, the following major themes were created from the qualitative interview data using the PIET-T model. The themes and subthemes are also summarised in online supplemental table 2.

### Theme 1: User-centred and organisation supported programmes (Population)

Regarding this theme, stakeholders discussed the health, well-being and activity services they were currently involved in, which included a diverse range of older adults.

#### Different programmes for diverse groups

Stakeholders reported that their organisation provided a range of physical activity programmes, including walking groups, exercise classes, yoga and an online activity programme to engage older adults to take part in various activities and make sure that activities were inclusive of individuals with a range of capabilities.

We try to arrange different activities for older adults, such as walking and low-level exercises (eg, chair-based exercise, yoga, and dance classes), to allow them to choose based on their ability. (Stakeholder 10, Male, Civil officer)

In my current job, I arrange different activities for multi-diverse older people with different needs. We try to engage them in different physical activities, different social activities just to make them more lively and more active. (Stakeholder 9, F, Engagement officer)

Numerous stakeholders also discussed that, along with their own organisation, they also work with other organisations that facilitate the involvement of older adults from ethnically diverse backgrounds.

We collaborate with local faith groups and cultural societies to ensure that older people from diverse backgrounds know about our organisations and can access our programmes. (Stakeholder 1, Male, Government officer)

We partner with the voluntary sector and local community groups to reach older adults from different ethnic backgrounds. (Stakeholder 3, Female, Research officer)

A few stakeholders also indicated language as a significant obstacle when working with ethnically diverse older adults, emphasising the significance of designing information and resources that are easily accessible to them:

I worked mainly with ethnic minority older adults, that is why language is a huge issue therefore we planned culturally appropriate communication strategies. (Stakeholder 2, Female, Programme lead)

#### Coproduced programmes

Stakeholders highlighted that their organisation co-produced programmes with older adults, which they reported made the programmes more effective and efficient in terms of timing and resource use. This approach reflects collaboration with older adults in both programme design and implementation.

…we created something tailor-made, co-produce programme with older people …that were much more effective, efficient in terms of timings and resources. (Stakeholder 1, Male, Government officer)

From the beginning, we try to involve older adults in planning the programme to ensure it is feasible and enjoyable and to maintain their engagement. (Stakeholder 4, Female, Research officer)

Stakeholders highlighted that their organisation prioritises physical activity to help older adults stay active, promote their health and well-being, and remain socially connected rather than isolated.

Our organization’s important focus is to keep older adults physically active to help them to remain independent and connected with the community. (Stakeholder 6, Female, Programme lead)

The majority of our sessions include some kind of physical activity since keeping older adults active is a key component of what we do. (Stakeholder 5, Male, Engagement officer)

Additionally, they explained that older adults are now more socially isolated due to the COVID-19 pandemic; therefore, there is a greater need for physical activity programmes:

…we were looking at the older people and because of the COVID-19 situation the older people were isolated at home so how could we make them more active? (Stakeholder 6, Female, Programme lead)

A few stakeholders also mentioned that they had gained funding for older adults’ physical activity initiatives; nonetheless, they found it difficult to involve older adults in different physical activity activities as shared by a participant.

…Last year we got some money …to set up the walking group… but the older group did not join the group…but still women did take part in the walk…and they went on for 6 weeks and then they also stopped. As soon as it was November and December, …they did not go for walking again. (Stakeholder 3, Female, Research officer)

### Theme 2: Intervention appropriateness and adaptations (Intervention)

The majority of the stakeholders viewed the intervention as practical for implementation in the real-world. They believed that the intervention would encourage older adults to be more active and reduce their time spent sitting down at home. Nonetheless, some cultural considerations and adjustments to the intervention component were recommended.

#### Feasible intervention

Stakeholders considered the intervention to be convenient and easy to implement as it was customised to meet the needs of older adults. They believed that the intervention might be successfully transferred to a wider audience and valued the concept of optimising older adults’ home space.

Yes, definitely, the intervention was easy to follow and can be replicated with a larger audience, I believe, because it was not difficult to implement. (Stakeholder 4, Female, Research officer)

This is a practical approach because it helps elderly people to stay active within their home environment instead of making any effort to move outside of home. (Stakeholder 10, Male, Civil officer)

Furthermore, these stakeholders believed that the feasibility of the intervention was positively influenced by the researcher’s support to the study participants.

I think it was easy in a way because it has a lot of instruction and guidance from the research team. (Stakeholder 1, Male, Government officer)

#### Cultural considerations

The significance of cultural factors in the adaptation of the intervention component was underlined as cultural limitations and significance may have an impact on the intervention’s recruitment and retention. Stakeholders shared that incorporating the intervention into the home was an appropriate and culturally relevant strategy, particularly for ethnically diverse older adults, as it recognised cultural preference for familiarity with private spaces and minimised challenges associated with accessing services outside of their home space.

Home-based intervention has suited participants as it was culturally sensitive, respected their privacy and reduced barriers to accessing services and increased their engagement. (Stakeholder 9, Female, Engagement officer)

Stakeholders also highlighted the role of family as an important socio-cultural factor in the recruitment and engagement of participants in the intervention. They proposed that families should be involved in the intervention itself, to support participation as well as co-delivering or engaging in activities with older adults.

I know it’s not easy to get out of your comfort zone and move if you are living a sedentary life; however, constant support of the family member will help them to take part in an intervention … It’s important to involve family members for the larger trial. (Stakeholder 7, Female, Programme lead)

Stakeholders discussed that translation of information into ethnically diverse languages will enhance the cultural relevance of the intervention and extend its reach to different inactive and sedentary older adults. As a result, they suggested using a variety of languages and formats for reminder messages. Concerns about language were also brought to light by the pamphlet and the sound messages that the devices produced as the written materials and audio messages do not use the native languages of the older adults. Ensuring that the materials are easily comprehended by the older adults is vital for effective communication and user engagement.

If text message is available in pictures or in different languages that might be good, rather than just written text. Could be a picture of somebody getting up in the different roles… (Stakeholder 9, Female, Engagement officer)

…the pamphlet you showed me I don’t feel that it was that much diverse. The images that you use that could be use in diverse format or diverse setting …Pakistani community or Chinese or African community members are operating. I think we need to make it more relevant to the community people. So, then people can resonate with the pamphlet more … For eg, …I am sitting down, and then suddenly I heard … You have to move” in the Bengali language… If it is somebody else’s language, you don’t associate it that well. (Stakeholder 6, Female, Programme lead)

Alongside the issues with languages, study participants notified a number of important aspects of the pamphlet layout that require attention. These include using larger font sizes and opting for a booklet format to improve readability and handling for older adult users.

…it should be in the booklet form. It should be big enough… (Stakeholder 2, Female, Programme lead)

#### Tailoring the programme

Given some older adults’ reluctance to use, and fear of, technology, they could be provided with greater flexibility in choosing the specific intervention component that they employ. Instead of offering older adults a multicomponent intervention that includes individualised health awareness sessions, wearable activity trackers, pamphlets and text messages, they might be allowed to choose the components that are in accordance with their needs and comfort levels. This customised approach would seek to increase recruitment, engagement and retention by meeting individual needs, which participants perceived could also assist in determining each component’s feasibility.

… you can provide elderly people with an option of selecting any particular component from the intervention …so they become more comfortable in taking part in research if they have any device related fear. (Stakeholder 10, Male, Civil officer)

Stakeholders also shared that older adults should be given a variety of technological options, enabling them to choose components according to personal preferences and confidence level:

I think comfort levels of older adults vary with technology, so intervention should offer different choices like wearable technology, apps, or smartphone alerts to permit them to select the method that best suits their preferences and comfort. (Stakeholder 9, Female, Engagement officer)

#### Modifications to intervention components

Stakeholders proposed a few modifications to the intervention’s components to meet the need of older adults, which they perceived would increase the effectiveness of the intervention. These suggestions highlighted the need for extended sessions to sustain behaviour change during the intervention (ie, several healthcare coaching sessions) as well as follow-up sessions after the intervention to record participants’ adherence following the completion of the intervention.

So perhaps maybe more session throughout the 12 weeks may be helpful just to make sure that people are on track and stay engaged with the programme. (Stakeholder 7, Female, Programme lead)

…there is a need for any kind of follow-up to check if they are maintaining what they have done or if they are still back to TV. (Stakeholder 8, Male, Engagement officer)

Stakeholders proposed the use of mobile phone application alerts for wearable activity trackers; however, they also identified some of the key obstacles to mobile phone use. Thus, instead of using a mobile phone, several stakeholders recommend employing activity trackers with minor modifications.

You could develop a mobile phone app… but again people don’t have mobile signals and Wi-Fi, so you have technologies accessibility issues. (Stakeholder 8, Male, Engagement officer)

…mobile phone you sometime leave it at your worktop, and you forgot and it’s not with you all the time. So, your device was sending vibration and was designed that they have to wear it around neck that was really good…maybe your item itself has to reduce in size. (Stakeholder 2, Female, Programme Lead)

### Theme 3: Organisational and system context (Environment)

The environment component assures the successful transfer and implementation of the intervention in a variety of contexts. The transferability of the intervention can be enhanced by an environment that fosters practice, reflection, feedback and collaboration with various service organisations.

#### Coordination and players; partners network

Support for funding was a key factor to begin, continue and transfer the intervention. However, many stakeholders discussed that due to organisational financial restrictions they would be unable to financially support the intervention and would instead be able to participate in transferability in other ways (eg, recruitment, retention and endorsement of the programme). These stakeholders suggested some funding sources, such as community service and non-governmental organisations, highlighting that they are crucial partners in the intervention’s transferability and their collaboration and support is fundamental to ensure that resources and support for the intervention are transferred efficiently.

There is also Swansea Voluntary Council, you can try them for funding, you can directly contact them… (Stakeholder 2, Female, Programme lead)

…it would be better if you plan all logistic with these organisations for intervention transferability. (Stakeholder 10, Male, Civil officer)

#### Local organisational setting; awareness and readiness

Stakeholders emphasised to optimise the intervention’s transferability, effective coordination with individuals holding leadership and decision-making roles within the organisation is necessary. Clear communication with leadership and senior management ensures that they are aware of the intervention and prepared to support its implementation.

Another thing, the coordination with those people at top hierarchy… and take them in loop for intervention transferability. (Stakeholder 7, Female, Programme lead)

#### Coordination with the healthcare system

Stakeholders mentioned that coordination of the broader healthcare system is necessary for the successful implementation of health interventions. However, scaling up and integrating the intervention across service delivery contexts requires a staged approach to demonstrate practicality and effectiveness.

If you work with public health practitioners, dietitians, and physiotherapists, this may be an easier route… You can identify how to recruit people. (Stakeholder 3, Female, Research officer)

…you need to a take a stages approach. Stage one: we have done this, now on stage two we need to scale up intervention and then stage three is the regional and then national level. (Stakeholder 1, Male, Government officer)

#### Policy and legislative alignment

For an intervention to be adopted at the regional and national levels, it must have an impact on or be consistent with national programmes and policies. Stakeholders emphasised that evidence of effectiveness is crucial to gain support, secure funding and ensure the intervention can be adapted according to policy requirements and local needs.

…to get to the bigger level, you should have more evidence to go to the national level and apply for funding. (Stakeholder 8, Male, Engagement officer)

### Theme 4: Transferability and implementation factors (Transferability)

It is crucial to understand how the intervention must be implemented in order to reach the target group by addressing various barriers and facilitators.

#### Barriers in transferability

As a potential barrier to the intervention’s transferability, the majority of stakeholders expressed concerns regarding the expenses associated with providing the technological device or activity trackers. Stakeholders agreed that resources are crucial to the implementation of the intervention. However, they acknowledged that their organisation lacks the resources necessary to expand the reach of this intervention.

Well, there is the cost associated for you guys to develop those devices; where is funding going to come for that? (Stakeholder 3, Female, Research officer)

I think the foremost challenge is lack of resources due to which we are unable to deliver this intervention. (Stakeholder 9, F, Engagement officer)

Another significant obstacle to implementing the intervention in practice, as identified by stakeholders, was team instability. They expressed concern that new hires who join a programme partway through the delivery of an intervention struggle to design and carry out programmes because they lack important background knowledge, which hinders the intervention’s progress. Stakeholders also highlighted that staff turnover disrupts team dynamics and that initiating new collaborative approaches requires time to build trust with participants. This loss of continuity may lead to participant withdrawal and negatively affect health outcomes, thereby restricting the transferability of the intervention.

As soon as I change role, my other colleagues also change the role and programme gets died [sic]… (Stakeholder 4, Female, Research officer)

The community members are let down and always face issues of passed on… Once you are being ill-treated or let down, then building that trust back is very difficult. (Stakeholder 6, Female, Programme lead)

#### Facilitators in transferability

Persistent support and assurance were seen as essential to encourage older adults’ participation. Stakeholders emphasised the importance of ongoing motivational support from staff to sustain older adults’ engagement with physical activity and sedentary behaviour interventions. Stakeholders suggested that embedding the intervention into pre-established programmes could strengthen existing services and improve current provision.

A staff needs to be constantly motivating them, pushing them and encouraging them. A continuous support because they are elderly people, they need constant support… (Stakeholder 10, Male, Civil officer)

It would be great if you merged your intervention with the on-going programme then elderly people would have dual benefit. (Stakeholder 9, Female, Engagement officer)

Establishing community involvement was thought to be a crucial component in transferring the programme to the real world. A number of stakeholders emphasised the necessity of sharing ownership with the community to expand the intervention research and achieve sustainability. They mentioned ways to involve the community, such as in the development and execution of interventions and including community members as role models. Additionally, they viewed incentives as a crucial factor in benefiting the researcher, rewarding participants for their time and encouraging their involvement in various research practices.

One way that I think would work well is you can present the older people wearing devices who were in the pilot phase and show it to other older people …that would benefit older people and help in scaling up the programme. (Stakeholder 5, Male, Engagement officer)

I think yes, with my work with the council, any time we did any work, we always compensated with vouchers … it increases the engagement, and I think it should be there in the programme. (Stakeholder 7, Female, Programme lead)

Stakeholders also noted that developing strong relationships and trust with older adult communities requires a substantial amount of time, particularly where no prior relationships exist. This challenge can be lessened by coordinating with appropriate individuals, such as healthcare professionals and trainers to increase intervention uptake. In addition, stakeholders highlighted the potential benefits of engaging with volunteer and charity organisations, which are often better equipped to interact with and relate to older adults in real-world settings. Such partnerships may support the development of multiple strategies to facilitate the transfer of the intervention into broader contexts.

To make it sustainable and transferable, I guess you have to target like health professionals, GP services, where there is an influx of older people… I think also you need to bring a voluntary/charity organisation that works directly with different age groups. If your work is represented by organisation and you have positive feedback that would gain more trust and more people… (Stakeholder 2, Female, Programme lead)

The stakeholders underlined the need for staff training, which is typically required to determine whether physical activity interventions can be effectively transferred. Additionally, they proposed that training third-sector staff like volunteers would make them aware of the research and help the programme become more transferable and sustainable. Importantly, stakeholders also expressed the view that training is necessary to meet the culturally sensitive needs of the older adults which will help staff to understand their varied cultural backgrounds and customs.

If you train the third sector partners, they can provide ongoing support to ensure that older people are using the device and adhering to the intervention. (Stakeholder 4, Female, Research officer)

…I would say that, like, extensive training so that staff become aware of the culture-specific needs. The staff needs to be culturally sensitive. (Stakeholder 1, Male, Government officer)

## Discussion

The perspectives of stakeholders are an essential step in facilitating the transferability of the home space intervention to reduce sedentary behaviour and increase physical activity among sedentary older adults to ensure that an intervention is prepared for practical implementation. However, there is limited research that has identified stakeholder perspectives to inform an intervention’s transferability, particularly in creating home-based physical activity and sedentary behaviour interventions for sedentary older adults using the PIET-T model, which considers the population (P), intervention (I), environment (E) and transfer (T).^32^

The population theme in the PIET-T model showed that stakeholders’ organisations provided different physical activity programmes to older adults from ethnically diverse groups. They emphasised that because many older adults are sedentary and socially isolated, there is a greater demand for physical activity programmes, especially following the COVID-19 pandemic.^40^ Moreover, it has been difficult to re-engage older adults in physical activity programmes due to the disturbance of their regular activity schedules, which affects their motivation and opportunity to be physically active.^41^ Some stakeholders highlighted the importance of creating customised interventions, enabling older adults to equally participate in research and the development of intervention strategies, an approach using coproduction that has aided them in effectively carrying out intervention implementation.^42^

Regarding the PIET-T model’s intervention component, stakeholders found the suggested home space intervention to be appealing. It meets the demands of older adults by offering practical and simple solutions, promoting light physical activity within their home setting. Furthermore, the wearable activity trackers produced automatic sound reminders and vibrations, customising the intervention to each individual’s behaviour, which were considered to be practical for ethnically diverse older adults. This is also aligned with participants’ views from earlier studies.^43 44^ They stated that the programme’s flexibility and convenience allowed them to remain active, helping them in reducing their sedentary time and incorporating activity into their daily routine.

The stakeholders proposed several changes to the home space intervention to enhance its implementation in practical contexts. The changes and alterations were recommended with the goal of fostering cultural appropriateness within the intervention. It was recommended that the content of the intervention (such as messages and pamphlet material) is translated into the native language that will promote older adults’ connection with the intervention. Additionally, it was also suggested to increase the number of health coaching sessions and adding a follow-up for long-term behavioural modifications. Previous research also suggested that incorporating a follow-up is important to monitor intervention adherence.^45 46^ In a similar vein, stakeholders suggested structural changes in the wearable activity tracker to make it more age friendly. This is consistent with earlier research that has identified that older adults are willing to use technology as long as it is user friendly.^43^

The environment component of the PIET-T model promotes collaboration with and assistance from other service groups to promote intervention transferability. In the current study, the majority of stakeholders expressed concerns regarding the funding needed for intervention delivery, similar to previous research,^5 32 47 48^ highlighting the necessity of collaboration with non-governmental organisations and community service organisations to support the intervention’s transferability. Research has also shown that third-sector funders must help to address the issue of underfunding for evidence-based health practices.^49^ Building trustworthy relationships with these organisations aids in navigating funding and arranging resources and support for the intervention transferability in various settings.

In the present study the intervention transfer in the PIET-T model was considered to be influenced by a wide range of factors, including staff instability and knowledge transfer. Stakeholders clarified that staff turnover within their organisation reduces the possibility of the transferability of the intervention, corresponding with previous findings.^50^ A number of stakeholders also underlined the necessity of training to facilitate knowledge transfer regarding the intervention and how it is delivered. This is consistent with earlier studies showing staff members need assistance and appropriate training^47 48^ to meet the needs of culturally diverse people to ensure an intervention’s transferability.^51^,^53^ Conversely, as a possible solution to these challenges, several stakeholders suggested using older adults as a valuable asset in transferability of the intervention.^5^ Their participation as role models may help to recruit participants to the intervention and provide them with the opportunity to take project ownership.^54^ Alongside this, community referral pathways and services also increase the intervention’s reach, they recommended involving a variety of stakeholders, including members of the third sector and healthcare professionals.^6^ Evidence has shown that community referral pathways (trainers and healthcare professionals) enhance the intervention uptake,^6^ helping to close the gap between healthcare and the community

Given the exploratory focus of this study, we sought representation across diverse stakeholders to obtain a range of perspectives. However, recognising that some groups (eg, government department stakeholders) were under-represented, the intent was to gain thematic saturation at overall stakeholder sample level instead of each stakeholder subgroup. We therefore interpret findings from under-represented stakeholder groups with caution. Also, the PIET-T model allowed for deeper exploration and provided rich insights in assessing and guiding the intervention transferability.^32^ Due to time and resource constraints, we are unable to include older adults in exploring the transferability process of intervention. However, we intend to conduct interviews with older adults in a larger effectiveness trial to assess the intervention’s real-world transferability. However, social desirability bias may arise as the primary researcher also designed the intervention and conducted interviews. Therefore, the researcher attempted to reduce bias by assuring participants that no personal identifiable information would be displayed or published; and by informing them to share their honest views, there were no right or wrong answers

## Conclusion

Exploring the perspectives of a diverse group of stakeholders was a crucial step in facilitating the transferability of the intervention and ensuring its readiness for implementation in real-world settings. The study findings informed several modifications to enhance the intervention’s applicability across contexts. These included translating study materials into participants’ native languages to improve accessibility, adjusting the duration of the intervention to better suit participants’ needs and providing alternative digital delivery options to accommodate varying levels of digital literacy and access. In addition, recommendations highlighted the importance of strengthening collaboration with local organisations and healthcare systems to support implementation and improve uptake among ethnically diverse older adults.

## Supplementary material

10.1136/bmjopen-2025-107380 online supplemental file 1

10.1136/bmjopen-2025-107380 online supplemental file 2

10.1136/bmjopen-2025-107380 online supplemental file 3

10.1136/bmjopen-2025-107380 online supplemental file 4

## Data Availability

Data are available upon reasonable request.
