# Supplementary material for: Stakeholder perspectives on transferability of a 12-week physical activity and sedentary behaviour intervention for ethnically diverse community dwelling older adults: a qualitative study
Source: BMJ Open. 2026 Apr 24;16(4):e107380. doi: 10.1136/bmjopen-2025-107380 (PMC13110531; doi:10.1136/bmjopen-2025-107380)
Supplement: online supplemental file 1 [file bmjopen-16-4-s001.pdf]

Supplementary file 1: COREQ 32---ITEM CHECKLIST

No. Item	Guide questions/description	Reported on Page #
Domain 1: Research team and reflexivity		
1. Inter viewer/facilitator	Which author/s conducted the interview?	Page # 8 (Data Collection)
2. Credentials	What were the researcher's credentials?	Page # 8 (Data Collection)
3. Occupation	What was their occupation at the time of the study?	Page # 8 (Data Collection)
4. Gender	Was the researcher male or female?	Page # 8 (Data Collection)
5. Experience and training	What experience or training did the researcher have?	Page # 8 (Data Collection)
6. Relationship with participants established	Was a relationship established prior to study commencement?	Page # 7 (Study recruitment and setting)
7. Participant knowledge of the interviewer	What did the participants know about the researcher?	Page # 7 (Study recruitment and setting)
8. Interviewer characteristics	What characteristics were reported about the inter viewer/facilitator?	Page # 8 (Data Collection)
Domain 2: study design		
9. Methodological orientation and Theory	What methodological orientation was stated to underpin the study?	Page # 5-7 (Study Design)
10. Sampling	How were participants selected?	Page # 7 (Study participants)
11. Method of approach	How were participants approached?	Page # 7 (Study recruitment and setting)
12. Sample size	How many participants were in the study?	Page # 8-10 (Result)
13. Non---participation	How many people refused to participate or dropped out? Reasons?	Page # 9 (Result)
14. Setting of data collection	Where was the data collected?	Page # 8 (Data Collection)
15. Presence of non---participants	Was anyone else present besides the participants and researchers?	NA
16. Description of sample	What are the important characteristics of the sample?	Page # 8 (Results)
17. Interview guide	Were questions, prompts, guides provided by the authors?	In-Depth Interview Guide (supplementary file 2)
18. Repeat interviews	Were repeat interviews carried out?	No
19. Audio/visual recording	Did the research use audio or visual recording to collect the data?	Page # 8 (Data Collection)
20. Field notes	Were field notes made during and/or	Page # 8 (Data Analysis)

	after the interview?	
21. Duration	What was the duration of the interviews	Page # 8 (Data Collection)
22. Data saturation	Was data saturation discussed?	Page # 8 (Data Collection)
23. Transcripts returned	Were transcripts returned to participants for comment and/or correction?	No
Domain 3: analysis and findings		
24. Number of data coders	How many data coders coded the data?	Page # 8 (Data Analysis)
25. Description of the coding tree	Did authors provide a description of the coding tree?	NA
26. Derivation of themes	Were themes identified in advance or derived from the data?	Page # 8-9 (Data Analysis)
27. Software	What software, if applicable, was used to manage the data?	NA
28. Participant checking	Did participants provide feedback on the findings?	No
29. Quotations presented	Were participant quotations presented to illustrate the themes/findings? Was each quotation identified?	Page # 9-16 (Results)
30. Data and findings consistent	Was there consistency between the data presented and the findings?	Page 16-19 (Discussion)
31. Clarity of major themes	Were major themes clearly presented in the findings?	Page # 9-16 (Results)
32. Clarity of minor themes	Is there a description of diverse cases or discussion of minor themes?	Page # 9-16 (Results)
