# Supplementary material for: Stakeholder perspectives on transferability of a 12-week physical activity and sedentary behaviour intervention for ethnically diverse community dwelling older adults: a qualitative study
Source: BMJ Open. 2026 Apr 24;16(4):e107380. doi: 10.1136/bmjopen-2025-107380 (PMC13110531; doi:10.1136/bmjopen-2025-107380)
Supplement: online supplemental file 2 [file bmjopen-16-4-s002.pdf]

TIDieR
Template for Intervention Description and Replication

The TIDieR (Template for Intervention Description and Replication) Checklist*:

Information to include when describing an intervention and the location of the information

Item number	Item	Where located ** Primary paper (page or appendix number)	Other ᵀ (details)
	**BRIEF NAME**		
**1.**	Feasibility of the behaviour modification intervention to minimise sedentary behaviour and enhance physical activity	______	______
	**WHY**		
**2.**	Importantly, previous research on OAs in this area has focused on activities that occur outside the home and there is a lack of research focusing on interventions that optimize home space environment This could pose a variety of challenges for OAs from ethnically diverse backgrounds, including financial difficulties, social responsibilities, insecurity, language barriers, a lack of resources, and restrictions based on religion and culture. This could be a contributing factor to the limited evidence regarding the effectiveness of interventions for increasing PA levels and decreasing SB among OAs from under-represented groups<br>Previous investigations focused on younger OAs who are at least 50 years old or 60 years old and they are not considered "older adults" using the established definition of people who are 65 years of age or older. Inclusion of younger adults in this research may have positively skewed the findings as younger people are more receptive to altering their behaviour and encounter fewer obstacles to lowering SB. Similarly, the most suitable behaviour change techniques (BCTs) for OAs (≥65 years) may differ from those for adults (50–64 years). Further, the demands and expectations regarding activity may differ depending on age group (for example, OAs may have distinct drives and abilities). Therefore, present study examined the feasibility of this multi-level intervention comprising a wearable activity tracker (WAT), coupled with a brief health coaching session, pamphlet and reminder messages to decrease SB and improve PA in the home environment in ethnically diverse OAs.	pg 5	______
	**WHAT**		
**3.**	The 12-week multicomponent intervention was designed to align to the principles of the socio-ecological model (SEM) and the habit formation model. The SEM emphasises the significance of taking into account the elements that may have an impact on PA and SB in the context of the built environment, such as the home setting. The intervention consists of 40–60 minutes face-to-face, personalised health coaching session, a pamphlet, and a WAT.	pg 5	______
**4.**	**WHAT**	pg 5	______

	Pre-post intervention PA was measured using a wrist-worn accelerometer (Actigraph wGT9X Link+, Pensacola, FL). The aim of the brief individualised health coaching session was to raise awareness of PA and SB and detrimental health consequences of inactivity and SB. The session emphasised how crucial it is to increase activity during the entire day and break up prolonged SB episodes within the home setting.<br>The pamphlet explained SB and PA, their effect on health, and recommended guidelines. They included hints/cues on utilizing the home environment to enhance activity and lower SB. For instance, suggesting a regular cue (for instance, when TV ads play) and a behaviour to perform in response to the cue (like stand up or move around your home).<br>The device sent alerts through vibrations and voice-associated sound messages (such as "Thrive for activity within home space"; "Move More— Sit Less" ; "The less I sit, the less stiff I get"; "Hustle for that muscle within the home"; "Tune your body into fitness," and "Keep calm and move within the home") after every hour of sedentary within the home space, acting as a cue or prompt to minimize extended sedentary time<br>During the daytime reminder messages were sent to participants via their mobile phones reminding them of their action plans and encouraging them to adhere to the intervention.		
	**WHO PROVIDED**		
**5.**	The intervention was delivered by the primary researcher	pg 5	
	**HOW**		
**6.**	The intervention consists of 40–60 minutes face-to-face, personalised health coaching session, a hard-copy of pamphlet, and a WAT and reminder messages via mobile.	pg 5	
	**WHERE**		
**7.**	The intervention was planned within the home space of the study participants	pg 5	
	**WHEN and HOW MUCH**		
**8.**	A personalised health coaching session consists of 40–60 minutes, hard copy of pamphlet to refer weekly wearable activity tracker need to wear for 12-weeks, reminder messages 3times/week.	pg 5	
	**TAILORING**		
**9.**	The 12-week intervention was planned to be adapted according to the combined knowledgebase from existing literature, Sports psychologist experts and shared perspectives of participants from study 1.<br>Length of intervention period was 12-week because **8–12 weeks intervention** helps to develop habit and maintain behaviour change while shorter interventions (e.g., 4–6 weeks) often exhibit short-term motivational benefits rather than long-term habit change. OAs received reminder messages three times per week throughout the intervention period. The activity tracker aimed to send vibrations and sound messages if participants is sedentary for an hour. The health coaching session was not repeated however participants had hard copies of pamphlet which they can refer.	pg 5	
	**MODIFICATIONS**		
**10.‡**	No modifications were done during the study		

	**HOW WELL**		
**11.**	Not Applicable.	pg 5	
**12.‡**	Actual: NA		

** **Authors** - use N/A if an item is not applicable for the intervention being described. **Reviewers** – use '?' if information about the element is not reported/not sufficiently reported.

† If the information is not provided in the primary paper, give details of where this information is available. This may include locations such as a published protocol or other published papers (provide citation details) or a website (provide the URL).
‡ If completing the TIDieR checklist for a protocol, these items are not relevant to the protocol and cannot be described until the study is complete.

* We strongly recommend using this checklist in conjunction with the TIDieR guide (see *BMJ* 2014;348:g1687) which contains an explanation and elaboration for each item.
* The focus of TIDieR is on reporting details of the intervention elements (and where relevant, comparison elements) of a study. Other elements and methodological features of studies are covered by other reporting statements and checklists and have not been duplicated as part of the TIDieR checklist. When a **randomised trial** is being reported, the TIDieR checklist should be used in conjunction with the CONSORT statement (see www.consort-statement.org) as an extension of **Item 5 of the CONSORT 2010 Statement.** When a **clinical trial protocol** is being reported, the TIDieR checklist should be used in conjunction with the SPIRIT statement as an extension of **Item 11 of the SPIRIT 2013 Statement** (see www.spirit-statement.org). For alternate study designs, TIDieR can be used in conjunction with the appropriate checklist for that study design (see www.equator-network.org).
