# Supplementary material for: Stakeholder perspectives on transferability of a 12-week physical activity and sedentary behaviour intervention for ethnically diverse community dwelling older adults: a qualitative study
Source: BMJ Open. 2026 Apr 24;16(4):e107380. doi: 10.1136/bmjopen-2025-107380 (PMC13110531; doi:10.1136/bmjopen-2025-107380)
Supplement: online supplemental file 4 [file bmjopen-16-4-s004.pdf]

Table 2: Themes, subthemes and quotations of study participants

Theme	Sub-theme	Description	Quotations
Theme 1: User-centred and Organisation Supported Programmes (Population)	Different programmes for diverse groups	Stakeholders reported that their organisation provided a range of physical activity programmes to make sure that activities were inclusive of individuals with a range of capabilities.	*'We try to arrange different activities for older adults, such as walking and low-level exercises (e.g., chair-based exercise, yoga, and dance classes), to allow them to choose based on their ability.' (Stakeholder 10, Male, Civil officer)*<br><br>*'In my current job, I arrange different activities for multi-diverse older people with different needs. We try to engage them in different physical activities, different social activities just to make them more lively and more active.' (Stakeholder 9, F, Engagement officer)*
	Co-produced programme	Stakeholders highlighted that their organisation co-produced programmes with older adults, which they reported made the programmes more effective and efficient in terms.	*'…we created something tailor-made, co-produce programme with older people …that were much more effective, efficient in terms of timings and resources.' (Stakeholder 1, Male, Government officer)*<br><br>*'From the beginning, we try to involve older adults in planning the programme to ensure it is feasible and enjoyable and to maintain their engagement.' (Stakeholder 4, Female, Research officer)*
Theme 2: Intervention appropriateness and adaptations (Intervention)	Feasible Intervention	Stakeholders considered the intervention to be convenient and easy to implement as it was customized to meet the needs of older adults. They believed that the intervention might be successfully transferred to a wider audience and valued the concept of optimizing older adults' home space.	*'Yes, definitely, the intervention was easy to follow and can be replicated with a larger audience, I believe, because it was not difficult to implement.' (Stakeholder 4, Female, Research officer)*<br><br>*'This is a practical approach because it helps elderly people to stay active within their home environment instead of making any effort to move outside of home.' (Stakeholder 10, Male, Civil officer)*
	Cultural considerations	Stakeholders shared that incorporating the intervention into the home was appropriate and culturally relevant strategy, as it recognized cultural preference for familiarity with private spaces.	*'Home-based intervention has suited participants as it was culturally sensitive, respected their privacy and reduced barriers to accessing services and increased their engagement.'*

		Stakeholders also highlighted the role of family as an important socio-cultural factor in the recruitment and engagement of participants in the intervention	*(Stakeholder 9, Female, Engagement officer)* *'I know it's not easy to get out of your comfort zone and move if you are living a sedentary life; however, constant support of the family member will help them to take part in an intervention … It's important to involve family members for the larger trial.' (Stakeholder 7, Female, Programme lead)*
		Stakeholders discussed that translation of information into ethnically diverse languages will enhance the cultural relevance of the intervention and extend its reach to different inactive and sedentary older adults. As a result, they suggested using a variety of languages and formats for reminder messages. Concerns about language were also brought to light by the pamphlet and the sound messages that the devices produced. The written materials and audio messages do not use the native languages of the older adults. Ensuring that the materials are easily comprehended by the older adults is vital for effective communication and user engagement.	*'If text message is available in pictures or in different languages that might be good, rather than just written text. Could be a picture of somebody getting up in the different roles...' (Stakeholder 9, Female, Engagement officer)* <br>*'…the pamphlet you showed me I don't feel that it was that much diverse. The images that you use that could be use in diverse format or diverse setting …Pakistani community or Chinese or African community members are operating. I think we need to make it more relevant to the community people. So, then people can resonate with the pamphlet more ... For e.g., …I am sitting down, and then suddenly I heard … You have to move" in the Bengali language... If it is somebody else's language, you don't associate it that well.' (Stakeholder 6, Female, Programme lead)*
	Tailoring the programme	Given some older adults' reluctance to use, and fear of, technology, they could be provided with greater flexibility in choosing the specific intervention component that they employ. Instead of offering older adults a multi-component intervention that includes individualised health awareness sessions, wearable activity trackers, pamphlets, and text messages, they might be allowed to choose the	*'… you can provide elderly people with an option of selecting any particular component from the intervention …so they become more comfortable in taking part in research if they have any device related fear.' (Stakeholder 10, Male, Civil officer)*

		components that are in accordance with their needs and comfort levels.<br><br>Stakeholders also shared that older adults should be given a variety of technological options, enabling them to choose components according to personal preferences and confidence level.	*'I think comfort levels of older adults vary with technology, so intervention should offer different choices like wearable technology, apps, or smartphone alerts to permit them to select the method that best suits their preferences and comfort.' (Stakeholder 9, Female, Engagement officer)*
	Modifications to intervention components	Stakeholders proposed a few modifications to the intervention's components to meet the need of older adults, which they perceived would increase the effectiveness of the intervention. These suggestions highlighted the need for extended sessions to sustain behaviour change during the intervention (i.e. several healthcare coaching sessions) as well as follow-up sessions after the intervention to record participants' adherence following the completion of the intervention	*'So perhaps maybe more session throughout the 12 weeks may be helpful just to make sure that people are on track and stay engaged with the programme.' (Stakeholder 7, Female, Programme lead)*<br><br>*'…there is a need for any kind of follow-up to check if they are maintaining what they have done or if they are still back to TV.' (Stakeholder 8, Male, Engagement officer)*
		Stakeholders proposed the use of mobile phone application alerts for wearable activity trackers however they also identified some of the key obstacles to mobile phone use. However, instead of using a mobile phone, several stakeholders recommend employing activity trackers with minor modifications.	*'You could develop a mobile phone app… but again people don't have mobile signals and Wi-Fi, so you have technologies accessibility issues.' (Stakeholder 8, Male, Engagement officer)*<br><br>*'…mobile phone you sometime leave it at your worktop, and you forgot and it's not with you all the time. So, your device was sending vibration and was designed that they have to wear it around neck that was really good…maybe your item itself has to reduce in size.' (Stakeholder 2, Female, Programme Lead)*

Theme 3: Organisational and system context (Environment)	Coordination and players; partners network	Support for funding was a key factor to begin, continue, and transfer the intervention. Stakeholders suggested some funding sources, such as community service and non-governmental organisations, highlighting that they are crucial partners in the intervention's transferability Therefore, stakeholders recommended that the researchers plan the implementation with such organisations to ensure that resources and support for the intervention are transferred efficiently.<br>establishing trustworthy connections with these organisations supports in finding financing resources and accomplishing the intervention's goal.	*'There is also Swansea Voluntary Council, you can try them for funding, you can directly contact them...' (Stakeholder 2, Female, Programme lead).*<br><br>*'…it would be better if you plan all logistic with these organisations for intervention transferability.' (Stakeholder 10, Male, Civil officer)*
	Local organisational setting; awareness and readiness	Stakeholders emphasized that for the intervention to be transferable to a higher-level effective coordination with individuals holding leadership and decision-making roles within the organization is necessary. Clear communication with leadership and senior management ensures that they are aware of the intervention and prepared to support its implementation.	*'Another thing, the coordination with those people at top hierarchy… and take them in loop for intervention transferability.' (Stakeholder 7, Female, Programme lead)*
	Co-ordination with the healthcare system	Stakeholders mentioned that co-ordination of the broader healthcare system is necessary for the successful implementation of health interventions. However, scaling up and integrating the intervention across service delivery contexts requires a	*'If you work with public health practitioners, dietitians, and physiotherapists, this may be an easier route… You can identify how to recruit people.' (Stakeholder 3, Female, Research officer)*

		staged approach to demonstrate practicality and effectiveness.	*'…you need to a take a stages approach. Stage one: we have done this, now on stage two we need to scale up intervention and then stage three is the regional and then national level.' (Stakeholder 1, Male, Government officer)*
	Policy and legislative alignment	For an intervention to be adopted at the regional and national levels, it must have an impact on or be consistent with national programmes and policies. Stakeholders emphasized that evidence of effectiveness is crucial to gain support, secure funding, and ensure the intervention can be adapted according to policy requirements and local needs.	*'…to get to the bigger level, you should have more evidence to go to the national level and apply for funding.' (Stakeholder 8, Male, Engagement officer)*
Theme 4: Transferability and implementation factors (Transferability)	Barriers in transferability	As a potential barrier to the intervention's transferability, the majority of stakeholders expressed concerns regarding the expenses associated with providing the technological device, or activity trackers. Stakeholders agreed that resources are crucial to the implementation of the intervention. However, they acknowledged that their organisation lacks the resources necessary to expand the reach of this intervention	*'Well, there is the cost associated for you guys to develop those devices; where is funding going to come for that?' (Stakeholder 3, Female, Research officer)*<br><br>*'I think the foremost challenge is lack of resources due to which we are unable to deliver this intervention.' (Stakeholder 9, F, Engagement officer)*
		Another significant obstacle to implementing the intervention in practice, as identified by stakeholders, was team instability. They expressed concern that new hires who join a programme partway through the delivery of an intervention struggle to design and carry out programmes because they lack	*'As soon as I change role, my other colleagues also change the role and programme gets died [sic]...' (Stakeholder 4, Female, Research officer)*<br><br>*'The community members are let down and always face issues of passed on… Once you are being ill-treated or let down, then building that trust back is*

		important background knowledge, which hinders the intervention's progress. Stakeholders also highlighted that staff turnover disrupts team dynamics, and that initiating new collaborative approaches requires time to build trust with participants. This loss of continuity may lead to participant withdrawal and negatively affect health outcomes, thereby restricting the transferability of the intervention.	*very difficult.' (Stakeholder 6, Female, Programme lead)*
	Facilitators in transferability:	Persistent support and assurance were seen as essential to encourage older adults' participation. Stakeholders emphasised the importance of ongoing motivational support from staff to sustain older adults' engagement with physical activity and sedentary behaviour interventions. Stakeholders suggested that embedding the intervention into pre-established programmes could strengthen existing services and improve current provision.	*'A staff needs to be constantly motivating them, pushing them and encouraging them. A continuous support because they are elderly people, they need constant support...' (Stakeholder 10, Male, Civil officer)*<br><br>*'It would be great if you merged your intervention with the on-going programme then elderly people would have dual benefit.' (Stakeholder 9, Female, Engagement officer)*
		Establishing community involvement was thought to be a crucial component in transferring the programme to the real world. A number of stakeholders emphasised the necessity of sharing ownership with the community to expand the intervention research and achieve sustainability. They mentioned ways to involve the community, such as involving the community in the development and execution of interventions and including community members as role models. Additionally, they viewed	*'One way that I think would work well is you can present the older people wearing devices who were in the pilot phase and show it to other older people ...that would benefit older people and help in scaling up the programme.' (Stakeholder 5, Male, Engagement officer)*<br><br>*'I think yes, with my work with the council, any time we did any work, we always compensated with vouchers … it increases the engagement, and I think it should be there in the programme. '(Stakeholder 7, Female, Programme lead)*

		incentives as a crucial factor in benefiting the researcher, rewarding participants for their time and encouraging their involvement in various research practices.	
		Stakeholders also noted that developing strong relationships and trust with older adult communities requires a substantial amount of time, particularly where no prior relationships exist. This challenge can be lessened by coordinating with appropriate individuals, such as health care professionals and trainers to increase intervention uptake. In addition, stakeholders highlighted the potential benefits of engaging with volunteer and charity organisations, which are often better equipped to interact with and relate to older adults in real-world settings. Such partnerships may support the development of multiple strategies to facilitate the transfer of the intervention into broader contexts.	*'To make it sustainable and transferable, I guess you have to target like health professionals, GP services, where there is influx of older people… I think also you need to bring voluntary / charity organisation who works directly with different age groups. If your work is represented by organisation and you have positive feedback that would gain more trust and more people...' (Stakeholder 2, Female, Programme lead)*
		The stakeholders underlined the need for staff training, which is typically required to determine whether physical activity interventions can be effectively transferred. Additionally, they proposed that training third-sector staff like volunteers would make them aware of the research and help the programme become more transferable and sustainable. Importantly, stakeholders also expressed the view that the training is necessary to meet the cultural-sensitive	*'If you train the third sector partners, they can provide ongoing support to ensure that older people are using the device and adhering to the intervention.' (Stakeholder 4, Female, Research officer)*<br><br>*'…I would say that, like, extensive training so that staff become aware of the culture-specific needs. The staff needs to be culturally sensitive.' (Stakeholder 1, Male, Government officer)*

		needs of the older adults which will help staff to understand their varied cultural backgrounds and customs.	
