# Supplementary material for: Stakeholder perspectives on transferability of a 12-week physical activity and sedentary behaviour intervention for ethnically diverse community dwelling older adults: a qualitative study
Source: BMJ Open. 2026 Apr 24;16(4):e107380. doi: 10.1136/bmjopen-2025-107380 (PMC13110531; doi:10.1136/bmjopen-2025-107380)
Supplement: Supplementary file 5 [file bmjopen-2025-107380.reviewer_comments.pdf]

PEER REVIEW HISTORY

BMJ Open publishes all reviews undertaken for accepted manuscripts. Reviewers are asked to complete a checklist review form and are provided with free text boxes to elaborate on their assessment. These free text comments are reproduced below.

ARTICLE DETAILS

Title (Provisional)

Stakeholder perspectives on transferability of a 12-week physical activity and sedentary behaviour intervention for ethnically diverse community dwelling older adults: A qualitative study.

Authors

Meghani, Naureen Akber Ali; Hudson (JH), Joanne; Stratton (GS), Gareth; Mullin, Jane

---

VERSION 1 - REVIEW

**Reviewer**	**1**
**Name**	**Wingood, Mariana**
**Affiliation**	**Wake Forest School of Medicine, Department of Implementation Science**
**Date**	**19-Jan-2026**
**COI**	**None**

Thank you for providing me with the opportunity to review this well written manuscript. I have some minor suggestions for improvement prior to accepting this manuscript. This comments primarily relate to streamlining the manuscript to focus on the main purpose/aims of this study. I did have two major concerns, one was related to the sample size and was that the quotes provided do not support the sub-themes narratives written by the authors.

Introduction:

- A very minor recommendation is to decrease the number of “therefore” in the introduction, particularly the sentence that uses it twice.
- As this manuscript and journal is read internationally, please provide a reference and example that supports and further explains the following statement “The UK government makes significant investments to change OAs' low PA and high SB trends.”
- Third paragraph in the introduction could be reduced and streamlined to state something such as: 1) There are programs to address low PA and high SB; 2)

These programs are not working for BMEs; and 3) Reasons they are not working include lack of stakeholder/key partner engagement in the design of the programs.

- Personally, I perceive the introduction to have great information, but I think it is too long and can lose readers. Therefore, I recommend going straight from the third paragraph to the fifth paragraph about the importance of engaging stakeholders/key partners and then a section on transferability.

Method

- Study design- I recommend removing details about the process utilized and instead focusing on the design itself, utilizing a checklist, and providing details about ethical considerations and approval of the study.
- After the study design description, I recommend presenting the details about the 12-week intervention. This way the reader knows the intervention before you go into the details about what you plan on examining related to this intervention.
- The details within the PIET-T subsection are great, but I wonder if the team can provide more information of how they operationalized this information for this project. For example, the authors could include a table that mentions the four operational domains of PIET-T- population, intervention, environment, and transfer and how they operationalized it for this study; and/or how their data/qualitative interview questions mapped onto the domains of PIET-T (I see that this is mentioned in the data collection-but it may be valuable to mention this earlier in the PIET-T as well).
- Study participants-provide description on what is meant by "diverse stakeholders" – are they diverse by experience, their roles, their ethnicity, their age, etc?
- Provide references for the previous work described in the study participation section.
- For the exclusion criteria- what if someone's work consists of 75% working with OAs and 25% other population groups- where they excluded?
- How were "key organizations" identified/what makes an organization "key"?
- The value of IDI's should be placed in the design and some of the procedures mentioned in the design sub-section should be included here.
- For the intervention-I highly recommend utilizing something such as the TIDieR (Template for Intervention Description and Replication) to ensure that the intervention can be replicated and or providing references to studies that have this information. Without it there are many gaps within the description.

Results

- The n seems very small to meet saturation, particularly since the goal was to get input from each type of key partner or stakeholder. In such cases, it is often required that saturation is met for each sub-group of key partners or

stakeholders. Thus, having only 1 government department stakeholder makes me concerned about the conclusions that are drown from that group of key partners or stakeholders. Related to this there are also a number of sub-themes that are only supported by one quote, therefore, I recommend adding a table that outlines key findings within each of the PIET-T domains an at least 2 quotes that support each of those key findings.

- For different programs for diverse groups sub-theme- the authors state that *"stakeholders reported that in order to make sure that activities were inclusive of individuals with a range of capabilities, their organization provided range of PA programs."* However, their quotes do not support this conclusion. The quotes simply state that they offer a number of programs, but they don't say why they provide a large group of programs. Similarly, they state that *"Numerous stakeholders also discussed that, along with their own organization they also work with other organizations that facilitates involvement of Oas from ethnically diverse backgrounds."* Yet, their quotes does not highlight that the working with organization facilitates involvement of OAs from ethnically diverse populations.
- For need-based program sub-theme-the authors stated that the "*stakeholders highlighted that their organisation prioritises PA because they want OAs to be active and to promote their health and well-being. Additionally, they stressed that their programs and intervention were tailored to the requirements and circumstances of the OAs."* Yet the quote used does not support that the organization priorities PAs or that the program was tailored to the requirements and circumstances of the OAs. Instead the quote highlights that they co-produced programs with older adults and then state that the programs were more effective and efficient in terms of timing and resources, meaning the effectiveness and efficiency related to resources and not circumstances of the OAs.
- Feasibility-The quote regarding the use of lanyards around the neck does not related to the outcome of feasibility, but instead the outcome of appropriateness and acceptability. Thus, it seems that this section only has one quote related to feasibility. It would be valuable to expand this section.
- Adaptations- It seems that this sub-theme spills into the next sub-theme of cultural considerations and since there are no supporting quotes, I recommend removing it.
- Cultural considerations-The quotes provided focuses more on "ideal formatting of pamphlets" particularly related to language, health-literacy, and visual impairments. I understand that language and material adaptation is an essential part of cultural considerations, but it is just one aspect.
- Tailoring the program- I think this is a really valuable sub-theme, but wonder if there is more than 1 quote that supports this sub-theme. If not, can it be tied to

modifications to interventions components and labeled as Recommended Modifications?

- Modifications to intervention components- the following statement is not supported by any quotes *“They felt that by making these adjustments, the intervention would be more widely accessible and more effective in reaching its goal.”* To me it also seems that the first two quotes provided focus on the same thing- additional sessions can results in more long-term changes.
- Motivation- the statement *“Successful intervention implementation was thought to be significantly facilitated by the staff's motivation and engagement”* is not supported by the quote, which highlights that staff needs to constantly motivate the OA.
- Adjunct program- the quote provided does not reflect transferability, it reflects benefits of enhancing existing programs.
- General recommendation-the description under each theme seems more operational than data driven. I recommend removing this from the results and focusing on the results itself.

General:

- I recommend decreasing the number of abbreviations used in this manuscript as it makes it harder to follow. For example, OA for older adults can be confused for osteoarthritis and thus recommend not using this abbreviation. Other examples are BME which is only used twice and IDIs which is used seven times. I would think of cultural considerations

---

**Reviewer**	**2**
**Name**	**Macdonald, Marilyn**
**Affiliation**	**Dalhousie University**
**Date**	**30-Jan-2026**
**COI**	**None**

Peer-Review for BMJ Open January 30, 2026

Stakeholder perspectives on improving the transferability of a 12-week intervention for ethnically diverse older adults: A qualitative study.

Thank-you for the opportunity to review this work on a topic of importance as the numbers of older persons rises around the world.

The manuscript lacks flow and organization and would benefit from an editorial review.

Title – suggest title modification for clarity on what this study is about

Stakeholder perspectives on transferability of a 12-week physical activity intervention for ethnically diverse community dwelling older adults: A qualitative study.

Key words – Rethink these so that they make the work discoverable

Strengths and limitations – rethink these as well as you revisit the data analysis and identification of themes.

Abstract

Introduction – line 4, is it language problems or fluency in English?

Line 8 – how stakeholders' perspectives are implemented in transferring the interventions into real-world contexts. Do you mean how stakeholder perspectives influence implementation initiatives? Are the participants the individuals implementing the intervention?

Line 10 – perspectives instead of perceptions.

Methods

Unclear if PIET-T is a model to guide those doing the implementation of the intervention or development of the intervention. Methods are missing here.

Results – If reflexive thematic analysis was used. What themes were identified? What are cultural equalities?

Methods and results need to be further developed to support and clearly articulate the conclusions.

Introduction

Description of the intervention, and findings should have its own section.

Methods

Is the decision-maker a stakeholder?

Study participants n=? Page 8 before it is clear who the study participants are.

Exclusion criteria – unclear

Results – page 8 paragraphs 1 and 2 are confusing. Did 5 or 10 stakeholders participate?

I understand the PIET model informed the exploratory study, however the findings are the themes and Population, Intervention, Environment and Transfer are not the themes but rather areas to guide the interview. Revisit the data and identify a theme that captures stakeholder perspectives. For example, looking at the data under population I see that interventions need to be developed among organizations with users, and adaptable. A possible theme may be "Adaptable, user-centred, organization supported activities". Reflect on this and apply across the dataset.

The paper begins with perspectives then switches to perceptions. Be consistent with terminology.

Once the analysis is complete with a set of themes then the discussion will need to be revised accordingly.

Conclusion – revise accordingly

References - #56 not sure why this is included if Braun & Clark Thematic Analysis is used.

VERSION 1 - AUTHOR RESPONSE

S. No.	Reviewer 1 Comments	Point by point response
	Introduction:	
	Thank you for providing me with the opportunity to review this well written manuscript. I have some minor suggestions for improvement prior to accepting this manuscript. This comments primarily relate to streamlining the manuscript to focus on the main purpose/aims of this study. I did have two major concerns; one was related to the sample size and was that the quotes provided do not support the sub-themes narratives written by the authors.	We sincerely thank the reviewers for their very thorough, thoughtful, and constructive review. We greatly appreciate the time and care taken to provide such detailed and helpful feedback. The reviewers' comments have been invaluable in improving the manuscript, and their suggestions have helped us make several meaningful revisions that have strengthened the clarity and overall quality of the paper.
	A very minor recommendation is to decrease the number of "therefore" in the introduction, particularly the sentence that uses it twice.	Thank you for your feedback. We have removed repeated words and revised the introduction section. The changes are highlighted on page no. 4, line no. 2-12.
	As this manuscript and journal is read internationally, please provide a reference and example that supports and further explains the following statement "The UK government makes significant investments to change OAs' low PA and high SB trends."	Thank you for your feedback.<br><br>We have rectified this issue and provided the reference. The changes are highlighted on page no. 4, line no. 2-3.
	Third paragraph in the introduction could be reduced and streamlined to state something such as: 1) There are programs to address low PA and high SB; 2) These programs are not working for BMEs; and 3) Reasons they are not working	Thank you for your feedback. We have removed repeated words and revised the introduction section. The changes are highlighted on page no. 4, line no. 2-12.

	include lack of stakeholder/key partner engagement in the design of the programs.	
	Personally, I perceive the introduction to have great information, but I think it is too long and can lose readers. Therefore, I recommend going straight from the third paragraph to the fifth paragraph about the importance of engaging stakeholders/key partners and then a section on transferability.	Thank you for your comment<br><br>We have revised introduction and started straight from the third paragraph to the fifth paragraph. The changes are highlighted on page no. 4, line no. 2-50 and page no. 5, line no. 1-11.
	Methods	
	Study design- I recommend removing details about the process utilized and instead focusing on the design itself, utilizing a checklist, and providing details about ethical considerations and approval of the study.	Thank you for your feedback. We have revised the method parts.<br><br>The changes are highlighted on page no. 5, line no. 14 – 22 and on page no. 9, line no. 36-41
	After the study design description, I recommend presenting the details about the 12-week intervention. This way the reader knows the intervention before you go into the details about what you plan on examining related to this intervention.	Thank you for your comment. We have included a summary of the intervention after the design section. Full details of the intervention are reported in our previous publication, for which the reference is provided. The changes are highlighted on page no. 5, line no. 24-34.
	The details within the PIET-T subsection are great, but I wonder if the team can provide more information of how they operationalized this information for this project. For example, the authors could include a table that mentions the four operational domains of PIET-T- population, intervention, environment, and transfer and how they operationalized it for this study; and/or how their data/qualitative interview questions mapped onto the domains of PIET-T (I see that this is mentioned in the data collection-but it may be valuable to mention this<br>earlier in the PIET-T as well).	Thank you for your feedback and relevant comments.<br><br>We have included a table that mentions the four operational domains of PIET-T-population, intervention, environment, and transfer and how they operationalized it for this study.<br><br>The changes are highlighted on page no.6, line no. 22.
	Study participants-provide description on what is meant by “diverse stakeholders”– are they diverse by experience, their roles, their ethnicity, their age, etc?	Thank you for your comment.<br><br>We have revised study participants section and changes are highlighted on page 7, line no. 18-25.
	Provide references for the previous work described in the study participation section.	Thank you for your feedback. We have revised study participants section and

		changes are highlighted on page 7, line no. 25.
	For the exclusion criteria- what if someone's work consists of 75% working with OAs and 25% other population groups- where they excluded?	Thank you for your critical feedback. We have clarified this in our eligibility criteria. Changes are highlighted on page 7, line no. 27 -28 & on page 7, line no. 42-43.
	How were "key organizations" identified/what makes an organization "key"?	Thank you for your feedback. We agree that the term "key organizations" was not sufficiently defined and could be unclear. In response, we have removed the term "key" and revised the text to clearly describe the study recruitment. Changes are highlighted on page 7, line no. 1-3.
	The value of IDI's should be placed in the design and some of the procedures mentioned in the design sub-section should be included here	Thank you for highlighting it. We have added details to data collection.<br>The changes are highlighted on page no. 5, line no. 14 – 22 and on page 8, line no. 2-29
	For the intervention-I highly recommend utilizing something such as the TIDieR (Template for Intervention Description and Replication) to ensure that the intervention can be replicated and or providing references to studies that have this information. Without it there are many gaps within the description	Thank you for highlighting the importance of the TIDieR (Template for Intervention Description and Replication) guidelines. We have provided reference to our previous publication, where the intervention is described in detail on page 5-line no. 33. In addition, we have included the completed TIDieR checklist as a supplementary file to ensure transparency and facilitate replication.
	Results	
	The n seems very small to meet saturation, particularly since the goal was to get input from each type of key partner or stakeholder. In such cases, it is often required that saturation is met for each sub-group of key partners or stakeholders. Thus, having only 1 government department stakeholder makes<br>me concerned about the conclusions that are drown from that group of key partners or stakeholders. Related to this there are also a number of sub-themes that are only supported by one quote, therefore, I recommend adding a table that outlines key findings within each of the PIET-T domains	We thank the reviewer for this important comment. Our aim was to achieve thematic saturation at the level of the overall stakeholder sample, rather than saturation within each stakeholder sub-group. The purpose of this study was to explore cross-cutting factors influencing intervention transferability across the system, rather than to generate in-depth, subgroup-specific conclusions for each category of stakeholder.<br>Given the exploratory and feasibility nature of this work, we sought representation across key stakeholder types to capture a breadth of

	an at least 2 quotes that support each of those key findings	perspectives, while recognising that some groups (e.g., government department stakeholders) were represented by fewer participants. We therefore interpret findings from under-represented stakeholders with caution and have clarified this as a limitation in the manuscript. The changes are highlighted on page no.18, line no. 12-17.
	For different programs for diverse groups sub-theme- the authors state that *"stakeholders reported that in order to make sure that activities were inclusive of individuals with a range of capabilities, their organization provided range of PA programs."* However, their quotes do not support this conclusion. The quotes simply state that they offer a number of programs, but they don't say why they provide a large group of programs. Similarly, they state that *"Numerous stakeholders also discussed that, along with their own organization they also work with other organizations that facilitates involvement of Oas from ethnically diverse backgrounds."* Yet, their quotes does not highlight that the working with organization facilitates involvement of OAs from ethnically diverse populations.	Thank you for this important comment. We appreciate you drawing our attention to this issue.<br><br>In response, we have carefully reviewed and revised the quotes presented under this sub-theme to ensure that they more clearly and directly support the interpretations made in the text. Changes are made to page 10, line no. 9-50 and page 28, line no.13-15
	For need-based program sub-theme-the authors stated that the *"stakeholders highlighted that their organisation prioritises PA because they want OAs to be active and to promote their health and well-being. Additionally, they stressed that their programs and intervention were tailored to the requirements and circumstances of the OAs."* Yet the quote used does not support that the organization priorities PAs or that the program was tailored to the requirements and circumstances of the OAs. Instead the quote highlights that they coproduced programs with older adults and then state that the programs were more effective and efficient in terms of timing and resources, meaning the effectiveness and	Thank you for this feedback.<br><br>In response, we have carefully reviewed and revised the theme title and quotes presented under this sub-theme to ensure that they more clearly and directly support the interpretations made in the text. Changes are made to page 10, line no. 43-50 and on page 11, line no. 1-18.

	efficiency related to resources and not circumstances of the OAs.	
	Feasibility-The quote regarding the use of lanyards around the neck does not relate to the outcome of feasibility, but instead the outcome of appropriateness and acceptability. Thus, it seems that this section only has one quote related to feasibility. It would be valuable to expand this section.	Thank you for this comment. We have expanded this sub-theme. Changes are made to page 11, line no. 35-47.
	Adaptations- It seems that this sub-theme spills into the next sub-theme of cultural considerations and since there are no supporting quotes, I recommend removing it.	Thank you for your feedback. We have removed this sub-theme.
	Cultural considerations-The quotes provided focuses more on “ideal formatting of pamphlets” particularly related to language, health-literacy, and visual impairments. I understand that language and material adaptation is an essential part of cultural considerations, but it is just one aspect.	Thank you for this comment. We have expanded this sub-theme. Changes are made to page 12, line no. 12-39.
	Tailoring the program- I think this is a really valuable sub-theme, but wonder if there is more than 1 quote that supports this sub-theme. If not, can it be tied to modifications to interventions components and labeled as Recommended Modifications?	Thank you for this careful reading of the manuscript and for highlighting this issue. In response to that we have added quotes in this sub-theme. Changes are made to page 13, line no. 1-18.
	Modifications to intervention components-the following statement is not supported by any quotes *“They felt that by making these adjustments, the intervention would be more widely accessible and more effective in reaching its goal.”* To me it also seems that the first two quotes provided focus on the same thing- additional sessions can results in more long-term changes.	Thank you for this feedback. We have revised the explanation in the section. Changes are made to page 13, line no. 20-25.
	Motivation- the statement *“Successful intervention implementation was thought to be significantly facilitated by the staff's motivation and engagement”* is not supported by the quote, which highlights that staff needs to constantly motivate the OA.	Thank you for this important observation. We agree that the original statement was not fully supported by the accompanying quote. In that regard we have revised the text. Changes are made to page 15, line no. 36-40.

	Adjunct program- the quote provided does not reflect transferability, it reflects benefits of enhancing existing programs.	Thank you for this helpful feedback. We agree that the quote did not adequately reflect transferability. Hence, we have removed the reference to transferability from this section and revised the text to ensure that the interpretation more accurately reflects the content of the quote. Changes are made to page 15, line no. 49-50 and on page 16, line no. 1-20.
	General recommendation-the description under each theme seems more operational than data driven. I recommend removing this from the results and focusing on the results itself.	Thank you for this thoughtful suggestion. We appreciate your observation and we have revised the result section.
	**General:**<br>- I recommend decreasing the number of abbreviations used in this manuscript as it makes it harder to follow. For example, OA for older adults can be confused for osteoarthritis and thus recommend not using this abbreviation. Other examples are BME which is only used twice and IDIs which is used seven times. I would think of cultural considerations	Thank you for this valuable suggestion. We agree that the use of multiple abbreviations may reduce readability. Therefore, we have carefully reviewed the manuscript and reduced the number of abbreviations used.

S. No.	Reviewer 2 Comments	Point by point response
	Stakeholder perspectives on improving the transferability of a 12-week intervention for ethnically diverse older adults: A qualitative study. Thank-you for the opportunity to review this work on a topic of importance as the numbers of older persons rises around the world.	Thank you for your helpful comments and suggestions. We have carefully addressed them in the revised manuscript, which has helped improve the paper.
	Title – suggest title modification for clarity on what this study is about	Thank you for your suggestion regarding the title. We have adopted the recommended title and updated the manuscript accordingly. Changes are made to page 1, line no. 3-4
	Key words – Rethink these so that they make the work discoverable.	Thank you for your feedback. We have revised the key words. Changes are made to page 1, line no. 30.
	Strengths and limitations – rethink these as well as you revisit the data analysis and identification of themes	Thank you for your feedback. We have revised Strengths and limitations. Changes are made to page 3, line no. 2-20.
	Abstract	
	Introduction – line 4, is it language problems or fluency in English?<br><br>Line 8 – how stakeholders' perspectives are implemented in transferring the interventions into real-world contexts. Do you mean how stakeholder perspectives influence implementation initiatives? Are the participants the individuals implementing<br>the intervention?<br><br>Line 10 – perspectives instead of perceptions.	Thank you for these detailed comments. We have carefully revised the Introduction to address each point:<br><br>Line 4: We have clarified the wording to specify "fluency in English".<br><br>Line 8: We have rephrased the sentence to clarify that we are referring to how stakeholder perspectives influence the implementation of interventions in real-world contexts.<br><br>Line 10: We have replaced "perceptions" with "perspectives" to more accurately reflect the intended meaning.<br>Changes are made to page 12, line no. 3-13.
	Methods<br>Unclear if PIET-T is a model to guide those doing the implementation of the intervention or development of the	Thank you for this important feedback. We agree that the original text did not clearly explain the role of the PIET-T model or provide sufficient methodological detail.

	intervention. Methods are missing here	Therefore, we have revised the section. Changes are made to page 12, line no. 15-24.
	Results – If reflexive thematic analysis was used. What themes were identified? What are cultural equalities?	Thank you for this comment. We have removed the term “cultural equalities” to avoid confusion. Additionally, we have revised the Results section to clearly present the themes which are now interpreted and organized according to the PIET-T model. This ensures that the findings accurately reflect the data and the framework used for analysis, improving clarity and alignment with the methodology. Changes are made to page 12, line no. 26-32.
	Methods and results need to be further developed to support and clearly articulate the conclusions	Thank you for this comment. We have revised conclusion section based on methods.
	Introduction<br>Description of the intervention, and findings should have its own section.	Thank you for your comment. We have included a summary of the intervention in the Method section. Full details of the intervention are reported in our previous publication, for which the reference is provided. The changes are highlighted on page no. 5 line no. 24-34.
	Methods<br>Is the decision-maker a stakeholder?	Thank you for your comment. We have now explicitly stated in the manuscript. The changes are highlighted on page no. 5, line no. 39-42.
	Study participants n=? Page 8 before it is clear who the study participants are.	Thank you for your feedback. We have revised the study participant’s section. The changes are highlighted on page no. 7, line no. 19-25.
	Exclusion criteria – unclear	Thank you for your feedback. We have revised the exclusion criteria. The changes are highlighted on page no. 7, line no. 42-43.
	Results – page 8 paragraphs 1 and 2 are confusing. Did 5 or 10 stakeholders participate?	Thank you for your feedback. We have revised the result section. The changes are highlighted on page no.9, line no. 43-46.
	I understand the PIET model informed the exploratory study, however the findings are the themes and Population, Intervention, Environment and Transfer are not the themes but rather areas to guide the interview. Revisit the data and identify a theme that captures stakeholder perspectives. For example, looking at the data under population I see that	Thank you for this insightful feedback. We have carefully considered your suggestions and revised the presentation of our themes to better reflect the stakeholder perspectives while remaining aligned with the PIET-T model. Specifically, we have renamed and reframed the themes.<br>• Theme 1: User-centred and Organisation Supported Programmes (Population).

	interventions need to be developed among organizations with users, and adaptable. A possible theme may be "Adaptable, user-centred, organization supported activities". Reflect on this and apply across the dataset. The paper begins with perspectives then switches to perceptions. Be consistent with terminology.	• Theme 2: Intervention appropriateness and adaptations (Intervention).<br>• Theme 3: Organisational and system context (Environment).<br>• Theme 4: Transferability and implementation factors (Transferability).<br>Additionally, we have standardized terminology throughout the manuscript to consistently use "perspectives" rather than alternating with "perceptions," improving clarity and cohesion.
	Once the analysis is complete with a set of themes then the discussion will need to be revised accordingly.	Thank you for this feedback. We have revised discussion.<br>The changes are highlighted on page no. 16-19.
	Conclusion – revise accordingly	Thank you for this feedback. We have revised conclusion.<br>The changes are highlighted on page no. 18, line no. 21-30.
	References - #56 not sure why this is included if Braun & Clark Thematic Analysis is used.	Thank you for this feedback. We have removed reference # 56.

VERSION 2 - REVIEW

Reviewer 2
Name Macdonald, Marilyn
Affiliation Dalhousie University
Date 27-Mar-2026
COI

Peer-Review BMJ Open March 26, 2026

Stakeholder perspectives on transferability of a 12-week physical activity intervention for ethnically diverse community dwelling older adults: A qualitative study.

Thank-you for the revisions undertaken with this work. Line numbering is confusing and therefore not used by me in this review.

Abstract

When identifying a study design the following is de rigeur

A qualitative exploratory study employing Thematic Analysis……………..immediately the reader is situated in relation to the anticipated methodology and methods.

Results

Change the header to "findings" which is appropriate for use with qualitative methodologies. *Users* is unclear and leaves the reader wondering "who" the users are. I expected to see the themes here.

Conclusion

See later comments and consider revising.

Introduction

In the title and abstract the terms ethnically diverse older adults is used. The second sentence uses the term adults from minority groups. Please revise for consistency.

Page 6 – Are the stakeholders (implementing the intervention) considered end users or is it the recipient of the intervention the end user? This distinction needs to be made.

PIET-T Model

What is the distinction between primary and target settings? What is outcome transferability?

Page 8 – Why ask participants about the appropriateness for other settings since this is about community dwellers?

Data Saturation

Braun & Clark address this specifically in their (2022) book. Suggest revising the existing statement to be coherent with B & C.

Data Analysis

Thematic, therefore, avoid the use of categories and sub-categories in favor of Themes and sub-themes.

Researcher reflexivity – Important and need to be much more concise.

Conclusion

Provide some indication of what is meant by necessary modifications and recommendations. The last two sentences leave the reader wondering somewhat. The second last may be removed and the last articulated more clearly.

VERSION 2 - AUTHOR RESPONSE

S. No.	Reviewer Comments	Point by point response
	Thank-you for the revisions undertaken with this work. Line numbering is confusing and therefore not used by me in this review.	We thank the reviewer for constructive feedback
	**Abstract**<br>When identifying a study design the following is de rigeur<br>A qualitative exploratory study employing Thematic Analysis………………immediately<br>the reader is situated in relation to the anticipated methodology and methods.	Thank you for your feedback. The changes are highlighted on page no. 3, line no.26-32.
	Results<br>Change the header to “findings” which is appropriate for use with qualitative methodologies. *Users* is unclear and leaves the reader wondering “who” the users are. I expected to see the themes here.	Thank you for your feedback. We have revised the information. The changes are highlighted on page no. 3, line no. 35-39.
	Conclusion<br>See later comments and consider revising.	Thank you for your feedback. The changes are highlighted on page no. 3, line no. 15.
	Introduction<br>In the title and abstract the terms ethnically diverse older adults is used. The second sentence uses the term adults from minority groups. Please revise for consistency.	Thank you for your feedback.<br><br>We have rectified this issue and provided the reference. The changes are highlighted on page no. 4, line no. 2-3.
	Page 6 – Are the stakeholders (implementing the intervention) considered end users or is it the recipient of the intervention the end user? This distinction needs to be made.	Thank you for your feedback. We have revised entire sentence. The changes are highlighted on page no. 5, line no. 6-8
	PIET-T Model<br>What is the distinction between primary and target settings? What is outcome transferability?	Thank you for your comment. We have mentioned details. The changes are highlighted on page no. 5, line no. 39-41 and on page no. 6, line no. 8-9.

	Page 8 – Why ask participants about the appropriateness for other settings since this is about community dwellers?	Thank you for your comment. We have revised details. The changes are highlighted on page no. 8, line no. 18-20.
	Data Saturation<br>Braun & Clark address this specifically in their (2022) book. Suggest revising the existing statement to be coherent with B & C.	Thank you for your feedback. We have revised the information. The changes are highlighted on page no. 8, line no. 2 and 3.
	Data Analysis<br>Thematic, therefore, avoid the use of categories and sub-categories in favor of Themes and sub-themes.	Thank you for your comment. We have revised the terms. The changes are highlighted on page no. 9, line no. 39-41.
	Researcher reflexivity – Important and need to be much more concise.	Thank you for your feedback. We have revised this part. The changes are highlighted on page no. 9, line no. 14-24.
	Conclusion<br>Provide some indication of what is meant by necessary modifications and recommendations. The last two sentences leave the reader wondering somewhat. The second last may be removed and the last articulated more clearly.	Thank you for your comment. We have revised conclusions and changes are highlighted on page 18, line no. 15-23.
